# Errata Corrige. Orthostatic Reactivity in Patients with Ischemic Stroke in the Chronic Period. http://dx.doi.org/10.3889/oamjms.2015.090

**DOI:** 10.3889/oamjms.2015.095

**Published:** 2015-08-21

**Authors:** Danche Vasileva, Daniela Lubenova, Marija Mihova, Kristin Grigorova-Petrova, Antoaneta Dimitrova

**Affiliations:** 1 *University “Goce Delchev”, Faculty of Medical Sciences, Shtip, Republic of Macedonia*; 2 *National Sports Academy, Physical Therapy and Rehabilitation, Sofia, Bulgaria*; 3 *Ss Cyril and Methodius University of Skopje, Faculty of Computer Sciences and Engineering, Skopje, Republic of Macedonia*

**Keywords:** Orthostatic reactivity, Kinesitherapy, Neurorehabilitation, Neurodevelopment treatment, Ischemic stroke, Chronic period

## Abstract

**AIM:**

This study aims to trace the influence of specialized kinesitherapeutic methodology (SKTM) on orthostatic reactivity in patients with ischemic stroke in the chronic period (ISChP).

**MATERIAL AND METHODS:**

An active orthostatic test is used for the evaluation of the orthostatic reactions. The arterial blood pressure and heart rate were defined in the 10 minutes of supine position, before and after 1, 5 and 10 minutes of active upright position. The orthostatic autoregulation is evaluated four times - at the beginning of the study, on the 10th day, on the 1st month and three months after the start of the KT. The classification by Thulesius was used to separate the patients into two groups depending on the type of their orthostatic reactivity.

**RESULTS:**

At the beginning of the study of infringements symptomatic type orthostatic reactivity (SOR) was observed in 24 patients and hypertensive type orthostatic reactivity (HOR) was observed in the remaining 32 patients. Once applied SKTM establish improvement of orthostatic autoregulation for the groups SOR and HOR at the 10th day and the 1st month with a level of significance p <0.05.

**CONCLUSION:**

The applied specialized kinesitherapeutic methodology continued later as an adapted exercise program at home, has significantly improved the orthostatic reactivity in patients with orthostatic dysregulation due to the ISChP.

## Introduction

Cerebrovascular diseases are a leading cause of mortality in the world along with cardiovascular disease and a leading factor for premature disability.

Therapeutic principles of primary prevention are: systematic study of patients with risk factors for early detection of asymptomatic pathology of extra- and intracranial larger brain arteries; early detection of asymptomatic high-grade carotid stenosis with a view to surgery; treatment of the risk factors and accompanying diseases, leading to cerebrovascular disease such as hypertension, diabetes, hyperlipoproteinemia, cardiovascular disorders, nicotine dependence, alcohol consumption, etc.; changing the way and style of life with increasing physical activity, kinesitherapy, diet, weight loss, etc. [1].

The latest achievements in smartphone technology, including high processing power, storage, permanent internet connection, individualized methods of notification and proximity to customers, offer unique opportunities to use these technologies to improve health and strengthen research capacity globally [2].

Immediate and continuous implementation of effective and appropriate strategies for secondary prevention of patients after the first stroke or transient ischemic attack has the potential to reduce the burden of stroke to a quarter [3].

Orthostatic autoregulation is adaptive and compensatory mechanism against the gravitational redistribution of blood when switching from horizontal to upright posture. Changes in body position activated gravitational forces that cause an increase in intravascular pressure and redistribution of blood to the lower limbs in the so-called “hydrostatic indifferent point” [4]. Gravitational forces stimulate a complex of compensatory mechanisms to preserve a stable cerebral hemodynamics. An important role for limiting the orthostatic gravitational blood redistribution plays the “peripheral muscle pump”, i.e. the contraction of the calf muscles. Through the compression of the deep veins of the legs the blood returns to heart at the time of active standing and during physical exercise. The peripheral muscle pump insufficiency worsens the venous return to heart and contributes to the development of orthostatic intolerance and/or cerebral ischemia [5]. Physical exercise leads to changes in cerebral blood flow, which depends on the character, intensity and their duration. More than 25 years, systematic studies of Georgiev V, (1991) and Herholz K, et al, (1987) show that in the dynamic loads, without reaching tiredness, cerebral blood flow is increased with increasing intensity [6, 7].

In the last 10 years, studies in chronic stroke found that blood flow in the affected leg is significantly lower at rest [8, 9] and during exercise [10] compared to the intact limb. These unique unilateral adaptations which do not occur in undamaged young and older people, can affect the performance of daily activities and quality of life [11, 12]. Scientific studies show that lowering blood flow occurs secondary to decreased physical activity levels [11-13], which may affect the speed of blood flow, endothelial function and arterial diameter [14].

The role of kinesitherapy in the treatment of patients with ischemic stroke is well known, but its influence on orthostatic reactivity in patients with ischemic stroke is less studied. In literature in Bulgaria and Macedonia there are no research results to changes in orthostatic dysregulation in post-stroke patients. In a study of Lubenova D, et al, (2013, 2014), indicating that 6-month targeted physical therapy significantly improves orthostatic reactivity in patients with orthostatic dysregulation, due to diabetic polyneuropathy [15, 16].

The purpose of this study was to evaluate the effect of the specialized kinesitherapeutic methodology (SKTM) on orthostatic reactivity in patients with ischemic stroke in the chronic period (ISChP).

## Material and Methods

This study included 56 patients with ISChP (32 men and 24 women with right-sided 53.5%, 46.4% with left-sided hemiparesis, mean age 63.2 ± 8.8 years old, weight 77.9 ± 10.1 kg and height 169.2 ± 6.4 cm). All patients were selected on several criteria, and duration of disease from 5 months to 1 year which treatment course ends 3 months.

The clinical characteristics of the patients are given in Table 1, where the mean values, standard deviation, largest and smallest values of the descriptive characteristics are mentioned by: age, weight and height of patients studied, assessments of the stage of functional recovery according Brunnstrom and the test for spasticity according Ashworth, at the beginning of the study.

**Table 1:** Mean values, standard deviation, the highest and lowest values of descriptive characteristics by: age, weight and height of the patients studied, assessments of the stage of functional recovery according Brunnstrom and the test for spasticity according Ashworth, at the beginning of the study

Parameters	Age	Weight	Height	Brunnstrom - upper limb	Brunnstrom - lower limb	Ashworth - upper limb	Ashworth – lower limb
X±S_D_	63.2±8.8	77.9±10.1	169.2±6.4	4.2±0.7	4.8±0.6	1.6±0.6	1.1±0.5
Xmax	76	101	182	5	6	3	2
Xmin	40	60	152	3	4	1	0

For presence of a homogeneity in the study, patients were selected by the following criteria: have not severe respiratory insufficiency, cardiovascular insufficiency (third functional class), uncontrolled diabetes mellitus, cognitive and memory disorders, acute thrombophlebitis, severe decubital ulcer, severe orthopedic disorders impaired coordination and gait, ischemic heart disease, malignancies, severe progressive neurological disorders. The patients gave a written consent to participate in the study. All patients were able to move around alone or with help, and without serious problems in communication.

The drug therapy applied in researched patients by a neurologists included: Clopidogrel, Trombex, Enap, Corvitol, Exforge, Atacand, Renapril, Betalock zok), Germanium, Aspirin, Milgamma, Nergolin, Cavinton, Norvex, vit B complex, Nootropil, Plavix, Germanium, Fluoxitin, Metfogamma, Amaryl, Atoris, Rosvera, Agapurin. In different patients have different combinations of the above therapy.

The daily SKTM is of moderate intensity load. In the introductory part, the exercises were aimed to prepare the body for the upcoming exercises, gradual adaptation of the cardiovascular system (chest and diaphragmatic breathing). The main part of KT include therapeutic exercises for the transition from the occipital lying to standing, exercises for upper limb and control of the shoulder girdle, lower limb exercises and control of the trunk, pelvis and walking. The final part includes relaxation exercises to patients. After 10-day daily physical therapy, patients adapt program for home rehabilitation for three months [17].

Orthostatic autoregulation was evaluated using active orthostatic test at the beginning of the study, a 10-day, 1-months, and 3 months after the beginning of KT. The test was performed during the day, at room temperature between 21-23°C. Heart rate (HR), systolic (SBP) and diastolic (DBP) blood pressure were taken after 1, 5 and 10 minutes in the occipital lying position using a cuff method [5]. Then patients actively stand and the same measurements were performed after 1, 5 and 10 minutes active standing. Patients then return to the occipital lying position and register procedure is repeated again.

Depending on the type of orthostatic reaction our investigated patients are first divided according to the classification of Thulesius [18] into two groups: sympathicotonic orthostatic reactivity (SOR) where there is an increase of more than 20 beats/min for HR and decrease below 10 mmHg for SBP and hypertensive orthostatic reactivity (HOR) where there is an increase of more than 20 beats/min to HR and more than 10 mm Hg for SBP. This facilitates the interpretation of results as hemodynamic indicators are mixed in different groups.

### Statistical methods

The obtained data were processed statistically using descriptive analysis, variation analysis and alternative analyzes. Data are analyzed by Wilcoxan test and p-value less than 0.05 was considered statistically significant.

## Results

The results of the active orthostatic test in both groups of patients with SOR and HOR before treatment, the 10th day, 1st month and 3 months after kinesitherapy are summarized in Table 1 and Table 2, and the ratio between the obtained and baseline value of studied parameters and significance of changes in the patients studied are presented in Figure 1 and 2.

**Table 2:** Mean values, standard deviations, the largest and smallest data values of heart rate during orthostatic test in two (SOR and HOR) study groups before and after kinesitherapy distributed in classification by Thulesius

Parameter	Groups	SOR				HOR			
		at beginning	10^th^ day	1^st^ month	3^rd^ month	at beginning	10^th^ day	1^st^ month	3^rd^ month
Lying position 1									
1 min		81.4±6.1	79.5±5	78.5±5	78.5±5	85.6±9.6	84.6±6.4	83.3±6.4	83.3±6.4

	Xmax	88	88	88	88	96	92	90	90

	Xmin	70	72	70	70	65	72	70	70

5 min		80±5.1	77.5±4.8	76±4.9	76±4.9	83±8.8	82±6.6	79.5±5.7	79.5±5.7

	Xmax	86	85	84	84	94	90	88	88

	Xmin	72	70	69	69	63	68	68	68

10 min		79.6±4.9	77.1±4.3	75.6±5.1	75.6±5.1	82.4±9	81.6±7.4	79.1±5.9	79.1±5.9

	Xmax	86	80	84	84	94	90	88	88

	Xmin	82	70	68	68	62	65	67	67

Standing position									
1 min		105.5±5.9	95.5±6.2	88.7±4.9	88.7±4.9	109.7±8.8	103.9±6	92.8±7	92.8±7

	Xmax	111	108	98	98	119	115	100	100

	Xmin	95	86	82	82	90	94	78	78

5 min		100.6±6.8	91.3±6	85.8±4.6	85.8±4.6	102.9±9.8	98.8±6	88.3±6.1	88.3±6.1

	Xmax	108	102	94	94	115	108	96	96

	Xmin	90	80	80	80	88	88	76	76

10 min		98.5±7.3	89.9±6.1	84.3±4.5	84.3±4.5	101.3±10.1	97.2±5.2	86.7±6.8	86.7±6.8

	Xmax	106	102	90	90	110	106	94	94

	Xmin	88	80	80	80	85	88	72	72

Lying position 2									
1 min		92.1±7	84.3±3.4	79.6±4.6	79.6±4.6	94.2±7.6	92.1±6.3	82.9±7.2	82.9±7.2

	Xmax	100	96	88	88	100	98	90	90

	Xmin	82	74	72	72	75	78	68	68

5 min		87.8±8	4.3±6.2	77±5.1	77±5.1	89.5±7.8	86.5±7.2	79.9±6.4	79.9±6.4

	Xmax	98	88	86	86	96	95	86	86

	Xmin	72	70	70	70	70	70	65	65

10 min		86.8±7.7	78.5±5.3	76±4	76±4	88±7.5	84.8±6.2	78.8±6.1	78.8±6.1

	Xmax	96	84	82	82	96	90	86	86

	Xmin	72	69	70	70	70	70	65	65

**Figure 1:**
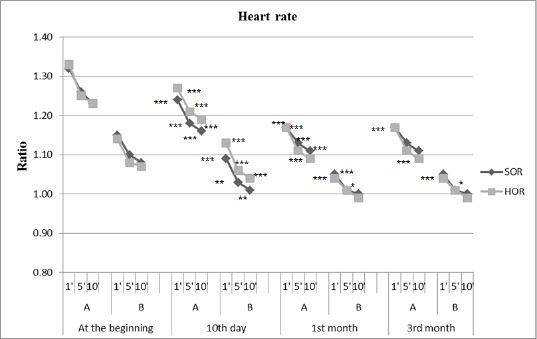
Changes in average heart rate during the active orthostatic test given as the ratio of the obtained results and baseline values; A = active state; B = lying position. * p <0.01, ** P <0.005, *** P <0.0001 = significant difference compared with baseline values.

**Figure 2:**
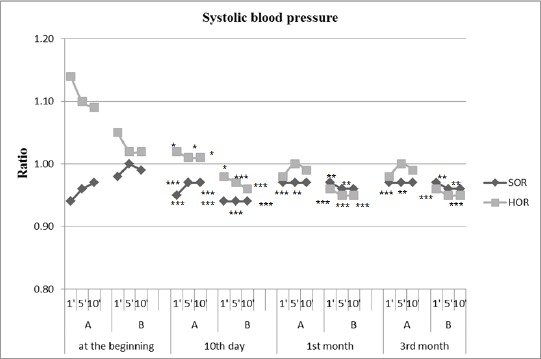
Changes in mean SBP during active orthostatic test given as the ratio of the obtained results and baseline values; A = active state; B = lying positio. * p <0.01, ** P <0.005, *** P <0.0001 = significant difference compared with baseline values.

When comparing the output data establishes that group HOR has higher values of heart rate in comparison with the group of SOR. This trend continued during the orthostatic test.

In the group of SOR is established significant normalization of HR in the first minute to the active standing. Compared to output a significant decrease in HR (from 25.0 to 18.0 beats/min) on day 10 of follow-up. A month after initiation of SKTM changes in HR during the active standing is expressed in the fact that the values of HR reduced to 13 beats/min, p <0.0001. There are similar changes in SBP. The initial orthostatic hypotension, objectified through a reduction in SBP in verticalisation, according to the values in 10 minutes occipital lying, is normalized after 10 days and is most significant increase in the 1st month (3.7 mm/Hg).

In group HOR is established a tendency towards normalization of orthostatic reactivity after application SKTM - HR is lowered significantly, according to baseline values in active standing, and SBP is stabilized around baseline values. Changes in HR are most marked in the 1st month, 13 beats/min, p <0.0001. Those changes to the SBP are most marked on the 10th day of the follow-up (increase in SBP is 3.5 mm/Hg, p <0.01). In the 1st month there is a tendency to reduce SBP, but this change was not statistically significant.

In both hemodynamic performances of the groups to 3 months, to establish such changes as in the 1st month, this means that SKTM has a long-term sustained impact.

## Discussion

Our study reveals that orthostatic reactivity in all patients with ISChP is improved after the applied SKTM. In the course of SKTM, continued later by performing the exercises at home, is normalized orthostatic reactivity in patients with ISChP and pathological orthostatic autoregulation. This is most clear in the 1st month from the beginning of the treatment, and then SKTM has a long-term sustained impact on hemodynamic parameters studied to the 3rd month.

The increase in blood pressure when standing increases α adrenergic responsiveness [19] of orthostatic stress, which leads to a marked increase in vascular resistance and arterial blood pressure, and may be due to, at least partly to the increase in baroreflex sensitivity. However, previous studies have reported that while resting sympathetic activity is lower in patients with orthostatic hypertension compared to those with orthostatic hypotension [19, 20].

**Table 3:** Mean values, standard deviations, the largest and smallest data values of systolic blood pressure during the orthostatic test in two (SOR and HOR) study groups before and after kinesitherapy, distributed classification by Thulesius

Parameter	Groups	SOR				HOR			
		At beginning	10^th^ Day	1^st^ Month	3^rd^ Month	At beginning	10^th^ Day	1^st^ Month	3^rd^ Month
Lying position 1									
1 min		141.6±8.6	135.8±5.8	131.2±5.9	131.2±5.9	147.1±9.9	144.6±10	138.7±7	138.7±7

	Xmax	155	145	140	140	160	155	145	145

	Xmin	130	130	120	120	130	125	130	130

5 min		139.1±7.4	132±5.6	128.7±3.6	128.7±3.6	143.6±9.5	140.5±10.2	136.9±7.1	136.9±7.1

	Xmax	150	140	135	135	155	150	145	145

	Xmin	125	125	120	120	128	120	125	125

10 min		140±7.2	132±5.6	128.7±5.5	128.7±5.5	144±9.8	140.5±10.2	136.3±6.9	136.3±6.9

	Xmax	150	140	135	135	155	150	145	145

	Xmin	125	125	125	125	128	120	125	125

Standing position									
1 min		131.2±10	126.6±7.8	125±7.2	125±7.2	165.1±9.4	144±15.6	134.9±12.4	134.9±12.4

	Xmax	145	135	135	135	175	165	155	155

	Xmin	110	115	115	115	150	115	120	120

5 min		135.4±10.7	128.7±6.6	125.8±4	125.8±4	158.7±8.3	143.1±12.1	136.8±9.1	136.8±9.1

	Xmax	155	140	130	130	170	155	150	150

	Xmin	120	120	120	120	145	120	125	125

10 min		136.2±10.4	128.7±6.6	125.8±4	125.8±4	158±9.4	143.1±12.1	135.6±10.9	135.6±10.9

	Xmax	155	140	130	130	170	155	150	150

	Xmin	120	120	120	120	140	120	120	120

Lying position 2									
1 min		137.5±6.7	125.4±5.6	125±4.6	125±4.6	151.9±8.8	137.8±11.5	131.2±8.3	131.2±8.3

	Xmax	145	140	135	135	165	150	145	145

	Xmin	125	120	120	120	135	120	120	120

5 min		140±6.2	125±4.6	124.1±3.5	124.1±3.5	147.8±8.3	136.5±10.1	130.9±8.6	130.9±8.6

	Xmax	150	135	130	130	155	150	145	145

	Xmin	130	120	120	120	130	120	120	120

10 min		139.1±6.5	125±4.6	124.1±3.5	124.1±3.5	147.1±8.3	135.9±10.6	130.3±9.2	130.3±9.2

	Xmax	150	135	130	130	155	150	145	145

	Xmin	130	120	120	120	130	120	120	120

Cerebral blood flow and systemic BP are positively associated [14], and its autoregulation is impaired in patients who have orthostatic tachycardia during an orthostatic challenge [21]. Moreover, postural change has been identified as the most important trigger of ischemic stroke out of seven predefined emotional, behavioral or environmental stimuli [22].

It has been shown that patients who have a greater orthostatic changes in blood pressure have higher plasma levels of brain natriuretic peptide and a wider distribution of silent cerebral infarction, suggesting cardiovascular overload in patients with hypertension [23].

The rehabilitation of patients with stroke invariably involves placing in the upright position and it is recommended that all stroke patients to be tested for orthostatic hypotension before each kinesitherapeutic program, especially those who are elderly, and those who have a more serious motor weakness and worse functional status, which is a predisposing factor for orthostatic hypotension.

The beneficial effect of kinesitherapy on orthostatic autoregulation, seen in our study, probably is due to different mechanisms. One of the patterns of changes in the central nervous system associated with brain reorganization is that there is a time window for the beneficial effects of the treatment effects. The improvement is greatest in the first 3-6 months, after which it stays or goes in some patients as functional recovery lifelong [24]. Yamomoto et al. [28] showed that exercise stimulates proprioceptive information, related to static posture, motor activity of human, realized through motor-visceral reflexes. It is well known that moderate intensity load stimulates the sympathetic nervous system, while in the recovery period after exercise dominates parasympathetic activity. Thus, during the orthostasis in healthy people with normal autonomous response is provided adaptive changes in systemic hemodynamics that maintain stable cerebral circulation and prevent orthostatic hypotension [25-28].

In conclusion, specialized kinesitherapeutic methodology (SKTM) continued later as exercise program at home during three months period, significantly improves orthostatic reactivity in patients with ischemic stroke in the chronic period and is supportive prolonged impact.
